# Electroactive Tissue Scaffolds with Aligned Pores as Instructive Platforms for Biomimetic Tissue Engineering

**DOI:** 10.3390/bioengineering2010015

**Published:** 2015-01-14

**Authors:** John G. Hardy, R. Chase Cornelison, Rushi C. Sukhavasi, Richard J. Saballos, Philip Vu, David L. Kaplan, Christine E. Schmidt

**Affiliations:** 1 Department of Biomedical Engineering, The University of Texas at Austin, Austin, TX 78712, USA; E-Mails: rcorneli@utexas.edu (R.C.C.); rushisukhavasi@gmail.com (R.C.S.); 2 J. Crayton Pruitt Family Department of Biomedical Engineering, University of Florida, Biomedical Sciences Building JG-53, P.O. Box 116131, Gainesville, FL 32611, USA; E-Mails: richardsaballos@live.com (R.J.S.); p.philip.v@gmail.com (P.V.); 3 Department of Biomedical Engineering, Tufts University, Medford, MA 02155, USA

**Keywords:** electroactive polymers, microfabrication, nerve guide, peripheral nerve, plastic electronics, topography

## Abstract

Tissues in the body are hierarchically structured composite materials with tissue-specific chemical and topographical properties. Here we report the preparation of tissue scaffolds with macroscopic pores generated via the dissolution of a sacrificial supramolecular polymer-based crystal template (urea) from a biodegradable polymer-based scaffold (polycaprolactone, PCL). Furthermore, we report a method of aligning the supramolecular polymer-based crystals within the PCL, and that the dissolution of the sacrificial urea yields scaffolds with macroscopic pores that are aligned over long, clinically-relevant distances (*i.e*., centimeter scale). The pores act as topographical cues to which rat Schwann cells respond by aligning with the long axis of the pores. Generation of an interpenetrating network of polypyrrole (PPy) and poly(styrene sulfonate) (PSS) in the scaffolds yields electroactive tissue scaffolds that allow the electrical stimulation of Schwann cells cultured on the scaffolds which increases the production of nerve growth factor (NGF).

## 1. Introduction

Bodily tissues are hierarchically structured composite materials with tissue-specific chemical and topographical properties, and these tissue-specific properties are known to act as cues (individually or in concert) that dictate the behavior of cells that inhabit them, and can potentially be engineered into instructional tissue scaffolds to achieve similar results [1,2,3,4,5,6,7,8,9,10,11,12,13,14,15]. Further to these cues, endogenous electric fields have been shown to act as behavioral cues during embryogenesis and wound healing, and devices that deliver exogenous electrical fields to the brain, ear or eye are already used in the clinic [4,16,17,18,19].

The complex interplay of chemical, electrical and topographical cues dictate the behavior of cells, and implantable biomaterials that act as instructional tissue scaffolds may facilitate the regeneration of functional tissues [2,7,8,12]. Topographical control of cell alignment is clearly observable within anisotropically aligned pores that are observed in bone [20,21], cardiac [22,23,24,25], nerve [3] and other tissues [6,7,26,27], which motivates the development of novel methodologies of imparting biomimetic porous structures within biomaterials.

While 3D printing technologies have prospects for the generation of porous materials, it is still challenging to fabricate porous structures that accurately mimic the chemical and intricate microscale and nanoscale topographical properties of native tissues, although solutions to these challenges are the subject of intense current research [28,29,30,31]. The removal of sacrificial templates embedded within a matrix (e.g., colloidal crystals, ice crystals, electrospun fibers) is another approach that allows the generation of hierarchically organized pore structures within materials [6,22].

**Figure 1:**
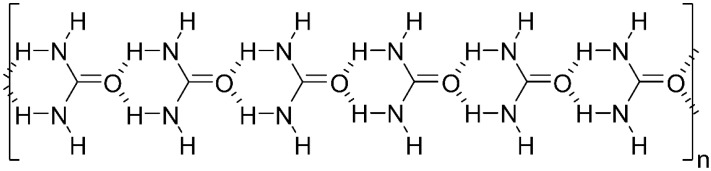
Hydrogen bond-mediated self-assembly of urea.

Urea is a cheap, non-toxic solid that self-assembles through hydrogen bonding interactions (Figure 1). The evaporation of water from aqueous solutions of urea yields dendritic crystals if performed uncontrolled, and seeds of urea crystals can initiate crystallization in a controlled manner prior to solvent evaporation to obtain less dendritic and relatively well aligned crystals over the length scale of a few hundred micrometers [32].

Zawko and coworkers reported the use of a sacrificial supramolecular polymer-based crystal template (urea) to impart pores [32] within photocrosslinkable biopolymer-based hydrogels that typically have mechanical properties similar to soft tissues such as those in the central nervous system [33]. Uncontrolled urea crystallization in aqueous solutions of photocrosslinkable biopolymers followed by crosslinking yielded hydrogels with dendritic pores within them [32]. Carefully controlling the seeding of urea crystal formation followed by crosslinking allowed the generation of pores that were less dendritic and relatively aligned over the length scale of a few hundred micrometers. Interestingly, fibroblasts cultured within the gels were observed to align parallel to the fibrillar microstructure of the hydrogels, whereas keratinocytes did not display any preferential orientation in response to the topographical cue [32].

Manufacturing materials using entirely aqueous processes, such as that described above is an appealing prospect, but it limits the selection of the materials used to those that are soluble in water (e.g., polysaccharides), and there are many devices used in the clinic based on polymers that are insoluble in water (e.g., PCL). Here we report the use of urea-based supramolecular polymer crystals as sacrificial templates in the preparation of porous tissue scaffolds from non-aqueous solvents. Moreover, we report a simple scalable methodology for aligning the supramolecular polymer crystals that allows the generation of aligned pores within the matrix of biodegradable polymer; and demonstrate that the pores serve as topographical cues to which cells respond by aligning. Generation of an interpenetrating network of PPy within the scaffold renders the scaffolds electroactive and facilitates the electrical stimulation of cells cultured on the scaffolds. These methodologies are broadly applicable to a variety of materials, and represent broad platform technologies for biomimetic tissue engineering.

Porous biomaterials are broadly applicable in tissue engineering [6,7,26,27,34], and we sought to demonstrate that electroactive materials generated using our methodology can electrically stimulate cells to yield a response [8,35,36,37,38,39,40,41,42,43,44,45,46]. Therefore, we used our methodologies to manufacture electroactive PCL-based scaffolds with biomimetic topographical properties, and showed that electrical stimulation of glial cells from the peripheral nervous system (*i.e*., Schwann cells) cultured on the scaffolds upregulated the production of nerve growth factor (NGF) which has been shown to promote peripheral nerve regeneration *in vivo* [47,48,49,50,51,52,53,54,55,56,57,58,59,60,61,62,63,64].

## 2. Experimental Section

### 2.1. Materials

Unless otherwise stated, all chemicals for synthesis and physicochemical analysis were of ACS grade, purchased from Sigma-Aldrich and used as received without further purification. Reagents for cell culture were purchased from Invitrogen (Carlsbad, CA, USA) unless otherwise noted. Neonatal rat Schwann cells isolated from sciatic nerves were purchased from ScienCell (Carlsbad, CA, USA).

### 2.2. Preparation of Polydimethylsiloxane (PDMS) Templates

**Figure 2:**
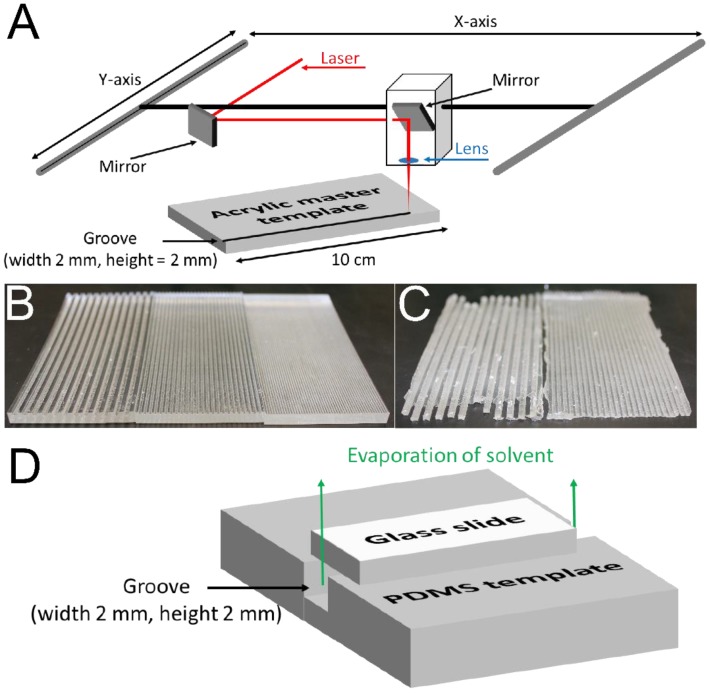
(**A**) Illustration of the experimental setup used to produce the hard acrylic master template with grooves with widths and heights of 2 mm. (**B**) Examples of the hard acrylic master templates produced: (**left**) grooves with widths and heights of 2 mm; (**center**) grooves with widths and heights of 1 mm; (**right**) grooves with widths and heights of 0.5 mm. (**C**) Examples of the flexible PDMS templates produced: (**left**) grooves with widths and heights of 2 mm; (**right**) grooves with widths and heights of 1 mm. (**D**) Illustration of the experimental setup using the flexible, grooved PDMS template covered with a glass slide, which facilitates controlled solvent evaporation and thereby preferential alignment of urea crystals within the grooves.

A 50 W laser cutter (Universal Laser Systems model VLS6.60, Scottsdale, AZ, USA) was used to manufacture acrylic master templates with grooves 2 mm in depth, 2 mm in width and 10 cm long (Figure 2A,B). Polydimethylsiloxane (PDMS) was prepared using a Sylgard^®^ 184 silicone elastomer kit in accordance with the manufacturer’s protocol. The PDMS precursors were mixed in a disposable plastic weigh boat using a disposable plastic 1 mL pipette tip prior to being slowly poured over the grooved acrylic templates housed in a disposable container of aluminum foil. Thereafter this setup was stored under vacuum in a desiccator for 4 days at room temperature to allow the PDMS to crosslink fully. The acrylic templates were cut out of the resulting PDMS films using a razor blade and the PDMS was peeled from the acrylic template allowing it to be reused (Figure 2C).

### 2.3. Preparation of PCL-Based Tissue Scaffolds with Aligned Pores

PCL (Mn 80 kDa, 1 g) and urea (1 g) were dissolved in hexafluoroisopropanol (10 mL). Samples were shaken in airtight centrifuge tubes (50 mL) at 1000 rpm using a Thermomixer C (Eppendorf International, Hauppauge, NY, USA) until the components had fully dissolved (typically 24 to 48 h). PDMS templates were placed on flat rigid surfaces, and optically clear hexafluoroisopropanol solutions of PCL/urea were pipetted into the grooves using disposable transfer pipettes. Glass microscope slides (width 2.5 cm, length 7.5 cm) were then placed on top of the PDMS templates (as depicted in Figure 2D) and the hexafluoroisopropanol was allowed to evaporate (typically 72 h), after which the slides were removed and the PCL/urea composites were placed in a container of water (100 mL). The samples were washed with water to remove the urea, exchanging the water every 3 h for 3 days, after which they were dried under high vacuum. The dimensions of the resulting white PCL-based tissue scaffolds—were determined using high precision digital calipers (ThermoFisher Scientific, Waltham, MA, USA). Samples were cut to lengths, appropriate for the various subsequent experiments, using a razor blade. The porosity of the samples can be calculated from:

$$Porosity\;\left(\%\right)=\left(\frac{volume\;of\;urea}{volume\;of\;PCL+\;urea}\right)*100$$

The samples had mass ratios 1:1 PCL:urea. The density of PCL is: 1.145 g/cm^3^, and 1 g therefore occupies 0.8733 cm^3^. The density of urea is: 1.32 g/cm^3^; so 1 g occupies 0.7575 cm^3^. Assuming that all of the urea is leached during the washing process, the porosity is: 46.4%.

### 2.4. Preparation of Electroactive PCL-Based Tissue Scaffolds with Aligned Pores

Pyrrole was purified by passage over basic alumina. White PCL-based tissue scaffolds with aligned pores were placed in disposable 50 mL centrifuge tubes containing a solution of pyrrole (291 µL, (84 mM), 1 eq.) and PSS (Mn 70 kDa, 0.799 g, (84 mM), 1 eq.) in distilled water (50 mL). Samples were sonicated for 5 minutes and cooled to 4 °C (for 1 h). Thereafter, ferric chloride (1.848 g, (228 mM), 2.7 eq.) was added. The samples were shaken to assure dissolution of the ferric chloride and then incubated for a further 24 h at 4 °C. Black electroactive tissue scaffolds with aligned pores were removed from the reaction mixture, placed in fresh distilled water, sonicated for 5 min, and then exhaustively washed (to remove monomers, oligomers and initiators) with deionized water until the water used to wash the materials was clear, colorless and the pH was neutral (*ca*. 48 h). Electroactive tissue scaffolds (PCL with an interpenetrating network of PPy and PSS) with aligned pores were dried under high vacuum at 21 °C. Samples were cut to lengths appropriate for the various subsequent experiments using a razor blade.

### 2.5. Scanning Electron Microscopy (SEM)

Images of porous PCL-based materials obtained using a scanning electron microscope (SEM). Samples were mounted on a SEM stub and sputter coated with Pt/Pd (15 nm) using a Cressington 208 benchtop sputter coater. All samples were imaged using a Zeiss Supra 40 VP field emission SEM.

### 2.6. Electrical Sheet Resistance

The electrical sheet resistance of the electroactive tissue scaffolds with aligned pores was measured in accordance with the method described by Schmidt [65] and Zhang [66]. In short, resistance (R in Ω) was measured between the two silver electrodes using a digital multimeter (DM-8A, Sperry Instrument, Milwaulkee, WI, USA). Sheet resistance (Rs) in Ω/square was calculated as follows:

$$R_{s}=RW/L$$

where W is the sample width (in cm) and L is the distance between the two silver electrodes (in cm). The electrodes were moved to different positions after each measurement, and the resistance R was recorded in at least ten different positions on the materials.

### 2.7. Fourier Transform Infrared Spectroscopy (FTIR)

Infrared spectroscopy was carried out on the samples to confirm that the surface chemistry of the scaffolds had changed after the growth of an interpenetrating network of PPy and PSS within the PCL matrix. A Thermo Scientific Nicolet 380 FTIR Spectrometer (Thermo Fisher Scientific Inc., USA) was used. Spectra were recorded in attenuated total reflectance (ATR) mode at 21 °C with a 1 cm^−1^ resolution and 128 scans (corrected for background and atmosphere using OMNIC software provided with the spectrometer). Samples were secured in position on the ATR crystal using the built-in clamp.

### 2.8. X-Ray Photoelectron Spectroscopy (XPS)

XPS was carried out on the samples to confirm that the surface chemistry of the scaffolds had changed after the growth of an interpenetrating network of PPy and PSS within the PCL matrix. XPS was performed on a Kratos Axis X-ray photoelectron spectrometer (Kratos Analytical Ltd., Manchester, UK). The binding energy was calibrated using the C 1s photoelectron peak at 284.6 eV as a reference. The CasaXPS computer program was used for peak fitting of the C 1s and O 1s peaks in the XPS spectra. The reported spectra are representative of two measurements at different positions on a sample.

### 2.9. In Vitro Degradation Study

Samples were incubated in PBS (1 mL) at 37 °C, in the absence or presence of cholesterol esterase (4 units/mL, Sigma Aldrich, USA). At specific time points the buffer was removed, the samples were carefully washed with deionized water. The samples were then dried under high vacuum to obtain a dry weight. The buffer (with or without enzymes) was replaced, and the mass of the film was recorded over a period of several days. Mass loss profiles represent the average of at least five samples.

### 2.10. In Vitro Cell Culture

#### 2.10.1. PCL-Based Tissue Scaffold Preparation and Sterilization

Commercially available tissue-culture treated Corning^®^ Costar^®^ tissue culture plates were used for control experiments. Non-electroactive and electroactive PCL-based tissue scaffolds with aligned pores were incubated in an aqueous solution of poly-D-lysine (PDL, 50 µg/mL) for 1 hour and then washed thoroughly with sterile water to remove any weakly adsorbed PDL (exchanging the water every 10 min for 1 h). Samples were inserted in untreated polystyrene tissue culture plates and sterilized by incubation in 70% ethanol followed by exposure to UV for 60 min.

#### 2.10.2. *In Vitro* Culture of Schwann Cells

After sterilization, scaffolds were incubated for 30 min under 3 mm of medium. Schwann cell growth medium was composed of: 25.5 mL of low glucose Dulbecco’s Modified Eagle Medium (DMEM); 8.5 mL of GIBCO^®^ Ham’s F-12 Nutrient Mixture; 350 µL Penicillin Streptomycin (1% of the final volume); 350 µL N2 supplement (2% of the final volume); Forskolin (5 µM); Neuregulin-1β (50 ng/mL). Medium was aspirated and replaced prior to Schwann cell seeding at 5000 cells/cm^2^ under 3 mm of medium, and incubated at 37 °C, 95% humidity, and 5% CO_2_. Cell viability before starting the experiment was determined by the Trypan Blue (Sigma, USA) exclusion method, and the measured viability exceeded 95% in all cases. After 2 days the medium was aspirated, the scaffolds were washed gently with PBS, and the cells were fixed with 4% paraformaldehyde in PBS for 15 min. The scaffolds were washed again with PBS (3 × 1 mL) and stored at 4 °C until they were stained and imaged.

#### 2.10.3. Electrical Stimulation of Schwann Cells

Electrical stimulation of Schwann cells was achieved employing a custom built setup. Non-conductive glass slides, polycarbonate wells (square polycarbonate blocks, thickness of 1 cm, sides of 2.5 cm, with square holes with sides of 0.9 cm cut out), [67,68] Dow Corning^®^ high vacuum grease, and medium binder clips (Staples^®^, Framingham, MA, USA) were sterilized by autoclaving. Holes were drilled into the sides of 10 cm polystyrene Petri dishes using a Dremel saw (Lowes, Mooresfield, NC, USA), and the plates were sterilized by exposure to UV for 60 min. Adhesive-backed copper tape (5 mm width, Ted Pella, Inc.), waterproof Kapton^®^ tape (1 cm width, Fisher Scientific, Waltham, MA, USA), wires and alligator clips were sterilized by exposure to UV for 60 min.

**Figure 3:**
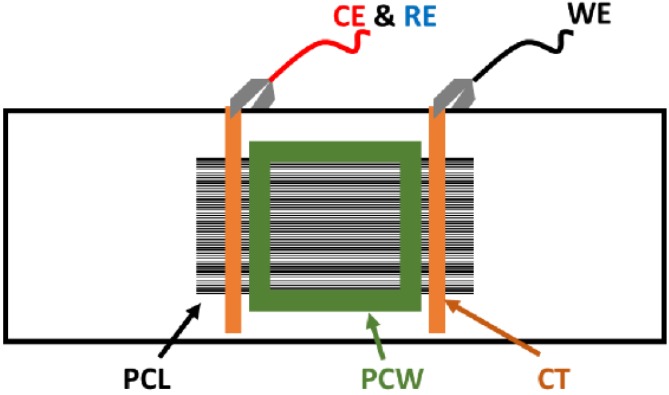
Experimental setup for electrical stimulation of electroactive PCL-based tissue scaffolds (Not to scale). (CE) counter electrode. (CT) copper tape. (PCL) electroactive PCL-based tissue scaffolds. (PCW) polycarbonate well. (RE) reference electrode. (WE) working electrode.

Electroactive PCL-based tissue scaffolds with aligned pores (prepared as described in Section 2.4) were placed on glass slides and secured in position with two thin strips of adhesive-backed copper tape that were attached to the films, parallel to one another and separated by a distance of *ca.* 4 cm. One face of the polycarbonate wells was coated with vacuum grease and placed on the electroactive tissue scaffolds, greased side down, in contact with the glass slide. A binder clip on either side of the well was used to secure this in position and render it water tight. A strip of copper tape was run between the parallel copper strips attached to the scaffolds and the ends of the slides as points of contact for the alligator clip-terminated wires attached to the multipotentiostat (CH Instruments, Austin, TX, USA). The counter and reference electrodes were connected together and clipped to copper tape on one side of the slide, and the working electrode was clipped to copper tape on the other side of the slide. Schwann cells were plated and cultured as described in Section 2.10.2. A potential step of +50 mV/mm was placed across the substrate for the duration of 1 h [69,70,71], after which the wires were disconnected and the substrates cultured as normal (see Figure 3).

#### 2.10.4. Fluorescence Staining and Imaging of Cells

Cells fixed with paraformaldehyde were permeabilized with 0.1% Triton X-100 (Fluka) and 2% bovine serum albumin (BSA) in PBS buffer for 5 min, followed by blocking with 2% BSA in PBS buffer for 30 min at room temperature. Actin filaments and cell nuclei within cells were stained with Alexa Fluor 488^®^ Phalloidin (Life Technologies, USA) for 30 min and 4,6′-diamidino-2-phenylindole (DAPI, Invitrogen, USA) for 5 min, respectively. The cells were thereafter washed three times with PBS and stored at 4 °C until images were acquired. Fluorescence images of cells were obtained using an Olympus IX70 inverted microscope equipped with an Olympus DP80 dual color and monochrome digital camera (a 1.4 megapixel Bayer mosaic color CCD camera) that was attached to the microscope with a 0.63 B-mount. Image Analysis was done using Olympus cellSens^®^ imaging software, Version 1.11.

#### 2.10.5. NGF Secretion Studies

Schwann cells were cultured under the conditions described above for 1 day, after which electrical stimulation for 1 h was optionally applied; non-stimulated controls included commercially available tissue-culture treated Corning^®^ Costar^®^ tissue culture plates, and non-electroactive/electroactive PCL-based tissue scaffolds with aligned pores. Medium was collected from the Schwann cell cultures immediately after electrical stimulation (0 h) and thereafter in intervals of 12 h for 3 days. The medium was stored at −20 °C for no more than 1 week prior to use in the Rat NGF ELISA Kit (Insight Genomics, Falls Church, VA, USA). ELISA kits were utilized in accordance with the protocol supplied with the kit, employing a Synergy HT Multi-Mode Microplate Reader (Biotek US, Winooski, VT, USA). Concentrations of NGF/mL were calculated based on standards supplied in the NGF ELISA Kit. Differences in cell numbers over the 3 day period of the assay were negligible as determined with an AlamarBlue^®^ cell viability assay, and concentrations of NGF in pg/mL are therefore presented uncorrected.

## 3. Results and Discussion

### 3.1. Preparation of PCL-Based Tissue Scaffolds with Highly Aligned Pores

We chose to produce tissue scaffolds incorporating aligned pores. The PDMS templates facilitated controlled solvent evaporation, preferential alignment of urea crystals within the grooves, and the resulting scaffolds to be easily removed from the templates to allow the sacrificial urea template to be removed by washing, yielding scaffolds with porosities of *ca*. 46.4%.

**Figure 4:**
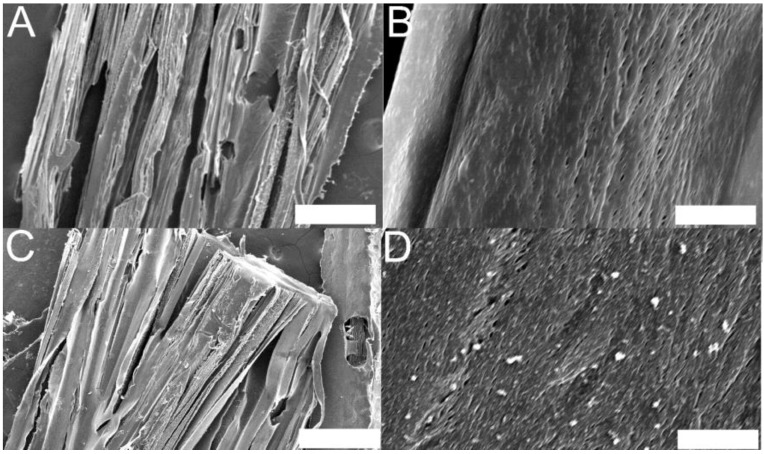
Scanning electron microscope images of sections of PCL-based tissue scaffolds with aligned pores. (**A**) Millimeter and micrometer scale topography of non-electroactive scaffolds, scale bar represents 500 µm. (**B**) Micrometer and nanometer scale topography of non-electroactive scaffolds, scale bar represents 10 µm. (**C**) Millimeter and micrometer scale topography of electroactive scaffolds, scale bar represents 500 µm. (**D**) Micrometer and nanometer scale topography of electroactive scaffolds showing evidence of increased nanometer scale surface roughness due to the presence of an interpenetrating network of PPy and PSS interwoven within the PCL matrix, scale bar represents 10 µm.

PCL-based tissue scaffolds produced using this methodology had thicknesses of *ca*. 0.4 mm, widths of *ca*. 2 mm, and pores (with widths of 10 s of micrometers as estimated by SEM, Figure 4A,B) aligned over lengths of up to 6.6 cm (*ca*. 88% of the length of the glass slide used to cover the grooves), which are clinically relevant length scales [8,72]. It is noteworthy that this would be straightforward to adapt to manufacture longer scaffolds using longer flexible templates and covers, and that this a very simple and inexpensive methodology well suited to laboratories across the world.

### 3.2. Preparation and Characterization of Electroactive PCL-Based Tissue Scaffolds with Aligned Pores

#### 3.2.1. Methodology for the Preparation of Electroactive PCL-Based Tissue Scaffolds

A simple method to reproducibly prepare electroactive PCL-based scaffolds was the generation of an interpenetrating network of electroactive PPy within the non-electroactive PCL matrix. The interpenetrating networks of PPy in PCL scaffolds were generated simply by incubating the scaffolds in aqueous solutions of pyrrole, PSS and ferric chloride for 24 h followed by exhaustive washing. [65,73] This simple process reproducibly produced mechanically stable electroactive tissue scaffolds with well-preserved micrometer and nanometer scale features; indeed, we found that use of higher concentrations of pyrrole (which is a good solvent for PCL) partially dissolved the PCL-based scaffolds, and extra crosslinking and washing steps were necessary to circumvent this problem (which also permits them to be sterilized by autoclaving) as reported by Yaszemski and co-workers [74,75,76,77]. Non-electroactive PCL-based scaffolds were white, whereas the electroactive scaffolds were black, and electron microscopy showed evidence of a slightly increased surface roughness on the nanometer scale due to the presence of polyelectrolyte complexes of positively charged PPy and negatively charged PSS interwoven with the PCL matrix (Figure 4C,D) [65].

#### 3.2.2. Spectroscopic Analysis of Non-Electroactive and Electroactive PCL-Based Tissue Scaffolds

**Figure 5:**
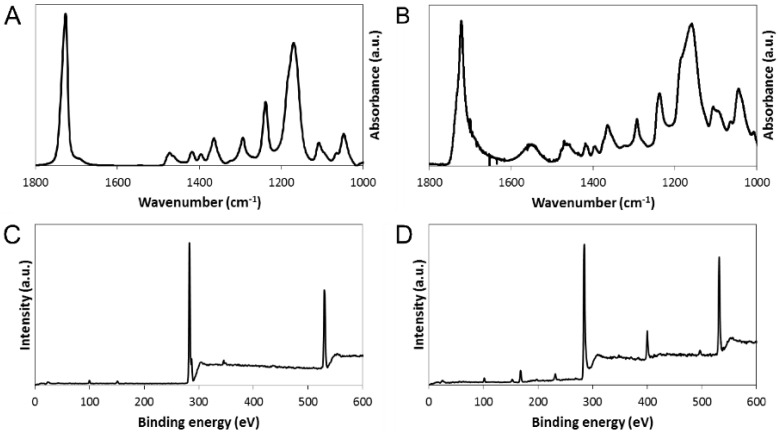
(**A** and **B**) FTIR spectra of PCL-based tissue scaffolds with aligned pores: (**A**) non-electroactive scaffolds, (**B**) electroactive scaffolds. Peaks observed at *ca.* 1543 and *ca*. 1480 cm^−1^ are characteristic of the antisymmetric and symmetric ring stretching modes of pyrrole [65,78]. (**C** and **D**) XPS spectra of PCL-based tissue scaffolds with aligned pores: (**C**) non-electroactive scaffolds, (**D**) electroactive scaffolds. Peaks at *ca*. 400 eV (N 1s) and *ca*. 168 eV (S 2p) are characteristic of PPy and PSS, respectively [65,73,79].

Comparison of infrared spectra recorded in ATR mode of the nonelectroactive scaffolds (Figure 5A) and the electroactive scaffolds (Figure 5B) confirmed the successful generation of an interpenetrating network of polyelectrolyte complexes of positively charged PPy and negatively charged PSS interwoven within the PCL matrix. Indeed, the peaks observed in the spectra of the electroactive scaffolds at *ca.* 1543 and *ca*. 1480 cm^−1^ are characteristic of the antisymmetric and symmetric ring stretching modes respectively (Figure 5B) [73,78,79]. In addition, comparison of X-ray photoelectron spectra of the nonelectroactive scaffolds (Figure 5C) and the electroactive scaffolds (Figure 5D) provided further evidence of alterations to the surface chemistry of the materials. The low intensity peak in the spectra at *ca*. 31 eV is the Na 2s and 2p peak (from NaCl), and the peak at *ca*. 500 eV is the corresponding Auger transition of sodium. The peak at *ca*. 99 eV is the Si 2p peak (a combination of the Si 2p1 and Si 2p3 peaks) from the underlying substrate. The peaks at *ca*. 285 eV correspond to C 1s, and the broad peak at *ca*. 531 corresponds to O 1s (a combination of C–O at *ca*. 531 and C=O at *ca*. 533) which all arise from the PCL backbone. The appearance of peaks in the spectra of the electroactive scaffolds at *ca*. 400 eV (N 1s) and *ca*. 168 eV (S 2p) are characteristic of PPy and PSS, respectively (Figure 5D) [65,66], confirming the successful generation of an interpenetrating network.

#### 3.2.3. Electrical Properties of Electroactive PCL-Based Tissue Scaffolds

Generation of an interpenetrating network of polyelectrolyte complexes of PPy and PSS within the nonelectroactive PCL-based scaffolds rendered them electroactive with sheet resistances of *ca*. 68 kΩ/square, on the order of analogous PPy-PSS polyelectrolyte complex-coated poly(lactic-*co*-glycolic acid) nanofibers (*ca*. 17 kΩ/square) [65] or PPy-heparin polyelectrolyte complex-coated Dacron^®^ 56 polyester fibers (*ca*. 16 kΩ/square) [66]. The relatively low resistance of the PPy-modified poly(lactic-*co*-glycolic acid) or Dacron^®^ 56 fibers is likely to be because the PPy is localized on the surface of the fibers [65], whereas the interpenetrating networks of PPy-PSS and PCL would have PPy-PSS in the bulk of the PCL and display some non-conductive PCL chains on the surface of the scaffolds [73].

#### 3.2.4. *In Vitro* Degradation of Non-Electroactive and Electroactive PCL-Based Tissue Scaffolds

**Figure 6:**
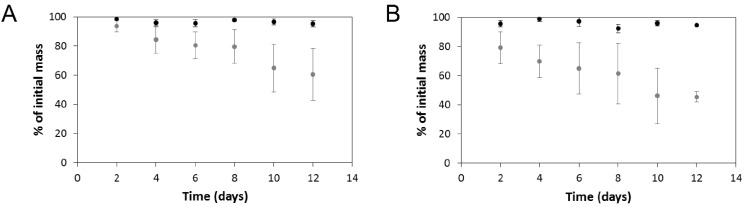
*In vitro* degradation profiles of the PCL-based tissue scaffolds in PBS. (**A**) Non-electroactive scaffolds: black circles in the absence of cholesterol esterase; grey circles in the presence of cholesterol esterase. (**B**) Electroactive scaffolds: black circles in the absence of cholesterol esterase; grey circles in the presence of cholesterol esterase. Error bars represent standard deviations.

While *in vitro* degradation experiments do not accurately reproduce conditions that materials encounter when implanted *in vivo* (particularly patient-specific immune responses or the tissue-specific distribution of enzymes), they are useful to confirm the potential of materials to degrade upon exposure to enzymes found *in vivo*. To demonstrate that enzymatic/hydrolytic degradation of the non-electroactive and electroactive PCL-based tissue scaffolds is possible, we incubated them in PBS in the absence or presence of a high concentration of an enzyme known to hydrolyze ester bonds in polyesters, cholesterol esterase (4 units/mL) [80,81,82,83,84]. When incubated in PBS for 12 days the masses of non-electroactive and electroactive scaffolds did not change significantly (Figure 6A,B, respectively) because hydrolysis of PCL occurs very slowly [85]. The presence of the esterase increases the rate of hydrolysis, resulting in a notable mass loss, *ca.* 40% over 12 days, from the non-electroactive PCL-based scaffolds (Figure 6A) and *ca*. 50% from the electroactive versions (Figure 6B). The presence of the electroactive polyelectrolyte complex of PPy and PSS appears to increase the hydrophilicity of the scaffolds allowing the enzyme to more easily access the PCL chains. The scaffolds are likely to degrade slowly if administered *in vivo* (over the period of several years) in line with other PCL-based materials [86] leaving behind the residual water insoluble polyelectrolyte complex of PPy and PSS that preclinical trials have shown to be relatively non-immunogenic. Indeed, histological analyses of tissue in the vicinity of polypyrrole-based tissue scaffolds implanted subcutaneously or intramuscularly in rats showed immune cell infiltration comparable to FDA-approved poly(lactic acid-co-glycolic acid) [87] or poly(D,L-lactide-co-glycolide) [87]. Similarly, there was no significant inflammation in the vicinity of polypyrrole-based materials implanted in the coronary artery of rats after 5 weeks [88], sciatic nerve guidance channels implanted in rats after 8 weeks [89], or electrodes in rat brains after 3 or 6 weeks [90].

### 3.3. In Vitro Cell Culture Studies on Instructional PCL-Based Tissue Scaffolds

**Figure 7:**
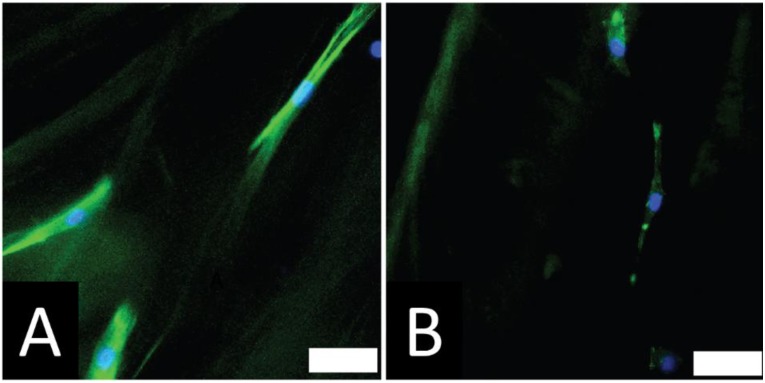
Cells respond to the topography of the PCL-based tissue scaffolds substrates and align on the substrates. (**A**) Schwann cells on non-electroactive scaffolds (scale bar represents 50 µm); (**B**) Schwann cells on electroactive scaffolds without electrical stimulation (scale bar represents 50 µm).

The anisotropic features are commonly observed in functional tissues (including bone, cardiac, musculoskeletal and nervous tissues), and scaffolds with biomimetic architectures perform well in the clinic [2,7,8,12]. We investigated the adhesion of rat primary Schwann cells on the non-electroactive and electroactive PCL-based tissue scaffolds with highly aligned pores. After 48 h in culture we observed that Schwann cells responded to the topographical cue by aligning preferentially with the long axis of the pores in the scaffolds (Figure 7), which is promising for future studies either with scaffolds derived from other polymers and using other cell types.

The role of endogenous electrical fields in the development of the nervous system motivated research into the application of exogenous electrical fields for therapeutic purposes, and preclinical studies show that electrical stimulation of damaged peripheral nerves for short periods of time (e.g., 1 h) improves their regeneration [18,69,70,71]. Electrical stimulation is known to affect cells differently depending upon a variety of factors including the cell type and species from which they were isolated, as discussed in detail in recent reviews [18,19]. Electrical stimulation of Schwann cells has been shown to increase the production of nerve growth factor (NGF) from Schwann cells cultured on electroactive indium tin oxide-based substrates [91], PPy-based substrates [92] or poly(3,4-ethylenedioxythiophene)-based substrates [93] or non-electroactive poly-L-Lysine-coated glass substrates [94]. NGF is a protein that plays a role in the growth, maintenance and survival of neurons. In fact, preclinical studies in rats showed that NGF promoted peripheral nerve regeneration [47,48,49,50,51,52,53,54,55,56,57,58,59,60,61,62,63,64], encouraging the development of NGF drug delivery systems [95], some of which are electrochemically triggered [96] and have the potential for regeneration of the nervous system.

**Figure 8:**
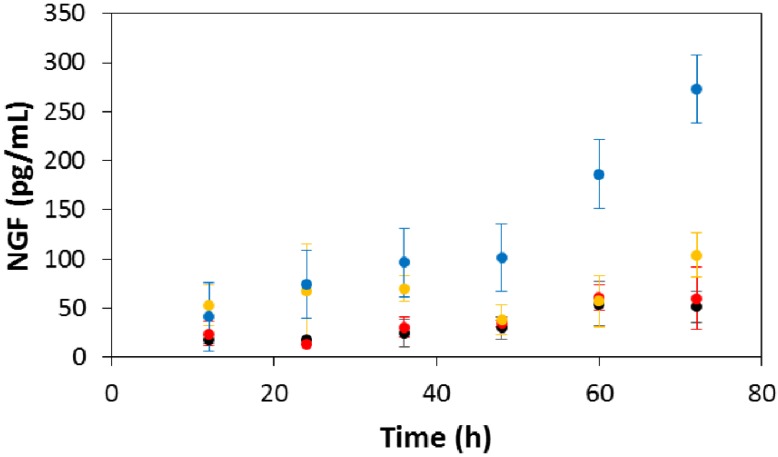
Concentration of Schwann cell-produced NGF in the culture medium. Black circles) commercially available tissue-culture treated Corning^®^ Costar^®^ tissue culture plates. (Red circles) non-electroactive PCL-based tissue scaffolds. (Yellow circles) electroactive PCL-based tissue scaffolds without electrical stimulation. (Blue circles) electroactive PCL-based tissue scaffolds with electrical stimulation. Error bars represent standard deviations.

Electroactive scaffolds such as those we report here clearly have the potential to act both as electrochemically-triggered drug delivery devices as well as instructive scaffolds that enable electrical stimulation of cells. We focused on the latter, investigating the amount of NGF expressed by rat Schwann cells when electrically stimulated (50 mV/mm) on the electroactive PCL-based tissue scaffolds with aligned pores, and non-stimulated controls including commercially available tissue-culture treated Corning^®^ Costar^®^ tissue culture plates, and non-electroactive/electroactive PCL-based tissue scaffolds with aligned pores. The concentration of NGF in the medium (in pg/mL) was determined using a Rat NGF ELISA Kit (Insight Genomics, Falls Church, VA, USA) immediately after electrical stimulation and thereafter in intervals of 12 h for 3 days. There were no significant differences in NGF production by Schwann cells in any of the non-stimulated controls over the 3 day study. In contrast to this, after 48 h in culture we observed that Schwann cells responded to the electrical cue and increased production of NGF to *ca.* three times the amount produced by an equivalent number of cells without electrical stimulation, a trend that was markedly more apparent during the following 24 h (Figure 8). Such increases in NGF production have been shown to encourage neurite outgrowth from neurons in a number of studies [97,98].

Our finding that cells respond not only to the topographical cue imparted through use of urea-based supramolecular polymer crystal sacrificial templates, but also to the electrical cue facilitated by an interpenetrating network of PPy shows the potential of our innovative biomaterials to function not only as scaffolds with potential for nerve regeneration, but also as platforms for the development of porous scaffolds for other tissues.

## 4. Conclusions

There is a need for biomaterials with biomimetic chemical and topographical properties for application as tissue scaffolds. Likewise, materials that facilitate the application of exogenous electrical fields have a variety of potential therapeutic applications. Here we present a novel process to manufacture instructional tissue scaffolds with biomimetic topographical properties and a process to render these scaffolds electroactive that allows electrical stimulation of cells cultured on them.

Sacrificial supramolecular polymer-based crystals composed of urea were used to generate pores within a matrix of a biodegradable polymer. We have developed a simple, inexpensive, scalable method of aligning the supramolecular polymer-based crystals within the biodegradable polymer matrix, allowing the preparation of scaffolds with macroscopic pores that are aligned over long, clinically relevant distances on the order of centimeters. The pores act as topographical cues to which rat Schwann cells responded by aligning.

We can prepare electroactive tissue scaffolds with biomimetic topographical properties using simple chemistry that allows the electrical stimulation of Schwann cells cultured on the scaffolds. This electrical cue increased the production of nerve growth factor (NGF) to more than three times the amount produced by non-stimulated cells, which may improve clinical outcomes during peripheral nerve regeneration.

Together, these simple, inexpensive methods represent a platform technology that facilitates the development of porous electroactive biomaterials.

## References

[1] Jaklenec A., Stamp A., Deweerd E., Sherwin A., Langer R. (2012). Progress in the tissue engineering and stem cell industry “are we there yet?”. Tissue Eng. B.

[2] Harrison R.H., St-Pierre J., Stevens M.M. (2014). Tissue engineering and regenerative medicine: A year in review. Tissue Eng. B.

[3] Wrobel M.R., Sundararaghavan H.G. (2014). Directed migration in neural tissue engineering. Tissue Eng. B.

[4] Balint R., Cassidy N.J., Cartmell S.H. (2013). Electrical stimulation: A novel tool for tissue engineering. Tissue Eng. B.

[5] Zhang B.G.X., Quigley A.F., Myers D.E., Wallace G.G., Kapsa R.M.I., Choong P.F.M. (2014). Recent advances in nerve tissue engineering. Int. J. Artif. Organs..

[6] Ma P.X. (2008). Biomimetic materials for tissue engineering. Adv. Drug Deliver. Rev..

[7] Lutolf M.P., Hubbell J.A. (2005). Synthetic biomaterials as instructive extracellular microenvironments for morphogenesis in tissue engineering. Nat. Biotechnol..

[8] Spivey E.C., Khaing Z.Z., Shear J.B., Schmidt C.E. (2012). The fundamental role of subcellular topography in peripheral nerve repair therapies. Biomaterials.

[9] Dvir T., Timko B.P., Kohane D.S., Langer R. (2011). Nanotechnological strategies for engineering complex tissues. Nat. Nanotechnol..

[10] Wang X., Yan Y., Zhang R. (2010). Recent trends and challenges in complex organ manufacturing. Tissue Eng. B.

[11] Griffith L.G., Swartz M.A. (2006). Capturing complex 3D tissue physiology *in vitro*. Nat. Rev. Mol. Cell Biol..

[12] Place E.S., Evans N.D., Stevens M.M. (2009). Complexity in biomaterials for tissue engineering. Nat. Mater..

[13] Fisher M.B., Mauck R.L. (2013). Tissue engineering and regenerative medicine: Recent innovations and the transition to translation. Tissue Eng. B.

[14] Salgado A.J., Oliveira J.M., Martins A., Teixeira F.G., Silva N.A., Neves N.M., Sousa N., Reis R.L. (2013). Chapter One—Tissue engineering and regenerative medicine: Past, present, and future. Int. Rev. Neurobiol..

[15] Berthiaume F., Maguire T.J., Yarmush M.L. (2011). Tissue engineering and regenerative medicine: History, progress, and challenges. Annu. Rev. Chem. Biomol. Eng..

[16] Hardy J.G., Lee J.Y., Schmidt C.E. (2013). Biomimetic conducting polymer-based tissue scaffolds. Curr. Opin. Biotechnol..

[17] Rutten W.L.C. (2002). Selective electrical interfaces with the nervous system. Annu. Rev. Biomed. Eng..

[18] Thompson D.M., Koppes A.N., Hardy J.G., Schmidt C.E. (2014). Electrical stimuli in the central nervous system microenvironment. Annu. Rev. Biomed. Eng..

[19] Yue Z., Moulton S.E., Cook M., O’Leary S., Wallace G.G. (2013). Controlled delivery for neuro-bionic devices. Adv. Drug Deliver. Rev..

[20] Despang F., Bernhardt A., Lode A., Dittrich R., Hanke T., Shenoy S.J., Mani S., John A., Gelinsky M. (2013). Synthesis and physicochemical, *in vitro* and *in vivo* evaluation of an anisotropic, nanocrystalline hydroxyapatite bisque scaffold with parallel-aligned pores mimicking the microstructure of cortical bone. J. Tissue Eng. Regen. Med..

[21] Spoerke E.D., Murray N.G.D., Li H., Brinson L.C., Dunand D.C., Stupp S.I. (2008). Titanium with aligned, elongated pores for orthopedic tissue engineering applications. J. Biomed. Mater. Res. A.

[22] Davidenko N., Gibb T., Schuster C., Best S.M., Campbell J.J., Watson C.J., Cameron R.E. (2012). Biomimetic collagen scaffolds with anisotropic pore architecture. Acta Biomater..

[23] Vejseli V., Lee E.J. Cardiac Fibroblast-Formed Anisotropic Decellularized Engineered Cardiac Tissues. Proceedings of 2013 39th Annual Northeast Bioengineering Conference (NEBEC).

[24] Kim D., Lipke E.A., Kim P., Cheong R., Thompson S., Delannoy M., Suh K.Y., Tung L., Levchenko A. (2010). Nanoscale cues regulate the structure and function of macroscopic cardiac tissue constructs. Proc. Nat. Acad. Sci. USA.

[25] Dunn D.A., Hodge A.J., Lipke E.A. (2014). Biomimetic materials design for cardiac tissue regeneration. WIRES Nanomed. Nanobiotechnol..

[26] Phillips J.B. (2014). Building stable anisotropic tissues using cellular collagen gels. Organogenesis.

[27] Nectow A.R., Kilmer M.E., Kaplan D.L. (2014). Quantifying cellular alignment on anisotropic biomaterial platforms. J. Biomed. Mater. Res. A.

[28] Zhu W., O’Brien C., O’Brien J.R., Zhang L.G. (2014). 3D nano/microfabrication techniques and nanobiomaterials for neural tissue regeneration. Nanomedicine.

[29] Sayyar S., Cornock R., Murray E., Beirne S., Officer D.L., Wallace G.G. (2014). Extrusion printed graphene/polycaprolactone/composites for tissue engineering. Mater. Sci. Forum.

[30] Ferris C.J., Gilmore K.G., Wallace G.G., Panhuis M.I.H. (2013). Biofabrication: An overview of the approaches used for printing of living cells. Appl. Microbiol. Biotechnol..

[31] Ferris C.J., Gilmore K.J., Beirne S., McCallum D., Wallace G.G., Panhuis M.I.H. (2013). Bio-ink for on-demand printing of living cells. Biomater. Sci..

[32] Zawko S.A., Schmidt C.E. (2010). Crystal templating dendritic pore networks and fibrillar microstructure into hydrogels. Acta Biomater..

[33] Seidlits S.K., Khaing Z.Z., Petersen R.R., Nickel J.D., Vanscoy J.E., Shear J.B., Schmidt C.E. (2010). The effects of hyaluronic acid hydrogels with tunable mechanical properties on neural progenitor cell differentiation. Biomaterials.

[34] Zohora F.T., Azim A.M.A. (2014). Biomaterials as porous scaffolds for tissue engineering applications: A review. Eur. Sci. J..

[35] Subramanian A., Krishnan U.M., Sethuraman S. (2009). Development of biomaterial scaffold for nerve tissue engineering: Biomaterial mediated neural regeneration. J. Biomed. Sci..

[36] Tresco P.A. (2000). Tissue engineering strategies for nervous system repair. Neural Plast. Regen..

[37] Saracino G.A.A., Cigognini D., Silva D., Caprini A., Gelain F. (2013). Nanomaterials design and tests for neural tissue engineering. Chem. Soc. Rev..

[38] Gu X., Ding F., Williams D.F. (2014). Neural tissue engineering options for peripheral nerve regeneration. Biomaterials.

[39] Geuna S., Gnavi S., Perroteau I., Tos P., Battiston B. (2013). Tissue engineering and peripheral nerve reconstruction: An overview. Int. Rev. Neurobiol..

[40] Marquardt L.M., Sakiyama-Elbert S.E. (2013). Engineering peripheral nerve repair. Curr. Opin. Biotechnol..

[41] Angius D., Wang H., Spinner R.J., Gutierrez-Cotto Y., Yaszemski M.J., Windebank A.J. (2012). A systematic review of animal models used to study nerve regeneration in tissue-engineered scaffolds. Biomaterials.

[42] Daly W.T., Knight A.M., Wang H., de Boer R., Giusti G., Dadsetan M., Spinner R.J., Yaszemski M.J., Windebank A.J. (2013). Comparison and characterization of multiple biomaterial conduits for peripheral nerve repair. Biomaterials.

[43] Hudson T.W., Liu S.Y., Schmidt C.E. (2004). Engineering an improved acellular nerve graft via optimized chemical processing. Tissue Eng..

[44] Hudson T.W., Zawko S., Deister C., Lundy S., Hu C.Y., Lee K., Schmidt C.E. (2004). Optimized acellular nerve graft is immunologically tolerated and supports regeneration. Tissue Eng..

[45] Hudson T.W., Evans G.R.D., Schmidt C.E. (2000). Engineering strategies for peripheral nerve repair. Orthop. Clin. North Am..

[46] Hudson T.W., Evans G.R.D., Schmidt C.E. (1999). Engineering strategies for peripheral nerve repair. Clin. Plast. Surg..

[47] Ikegami R. (1990). Changes of nerve growth factor (NGF) content in injured peripheral nerve during regeneration: Local synthesis of NGF by schwann cells. Nihon Seikeigeka Gakkai zasshi..

[48] Yu H., Peng J., Sun H., Xu F., Zhang L., Zhao B., Sui X., Xu W., Lu S. (2008). Effect of controlled release nerve growth factor on repairing peripheral nerve defect by acellular nerve graft. Chin. J. Repar. Reconstr. Surg..

[49] Sobue G. (1990). The role of schwann cells in peripheral nerve degeneration and regeneration—NGF-NGF receptor system. Clin. Neurol..

[50] Gambarotta G., Fregnan F., Gnavi S., Perroteau I. (2013). Neuregulin 1 role in schwann cell regulation and potential applications to promote peripheral nerve regeneration. Int. Rev. Neurobiol..

[51] Tonda-Turo C., Ruini F., Gnavi S., Di Blasio L., Primo L., Chiono V., Perroteau I., Ciardelli G. (2012). Naturally-derived hydrogels for growth factors release in peripheral nerve tissue engineering. J. Tissue Eng. Regen. Med..

[52] Scholz T., Rogers J.M., Krichevsky A., Dhar S., Evans G.R.D. (2010). Inducible nerve growth factor delivery for peripheral nerve regeneration *in vivo*. Plast. Reconstr. Surg..

[53] Wood M.D., Hunter D., Mackinnon S.E., Sakiyama-Elbert S.E. (2010). Heparin-binding-affinity-based delivery systems releasing nerve growth factor enhance sciatic nerve regeneration. J. Biomater. Sci. Polym. Ed..

[54] Kemp S.W.P., Webb A.A., Midha R. (2009). Sensorimotor analysis of peripheral nerve regeneration through T-tube chambers loaded with nerve growth factor (NGF). J. Peripher. Nerv. Syst..

[55] Valmikinathan C.M., Defroda S., Yu X. (2009). Polycaprolactone and bovine serum albumin based nanofibers for controlled release of nerve growth factor. Biomacromolecules.

[56] Yu H., Peng J., Guo Q., Zhang L., Li Z., Zhao B., Sui X., Wang Y., Xu W., Lu S. (2009). Improvement of peripheral nerve regeneration in acellular nerve grafts with local release of nerve growth factor. Microsurgery.

[57] De Boer R., Knight A.M., Wang H., Malessy M.J.A., Spinner R.J., Windebank A.J., Yaszemski M.J. (2008). Microsphere delivery of nerve growth factor (NGF) and glial cell line derived neurotrophic factor (GDNF) in supporting peripheral nerve regeneration in polymer scaffolds. Ann. Neurol..

[58] Kemp S.W.P., Walsh S.K., Midha R. (2008). Growth factor and stem cell enhanced conduits in peripheral nerve regeneration and repair. Neurol. Res..

[59] Lee A.C., Yu V.M., Lowe J.B., Brenner M.J., Hunter D.A., Mackinnon S.E., Sakiyama-Elbert S.E. (2003). Controlled release of nerve growth factor enhances sciatic nerve regeneration. Exp. Neurol..

[60] Aizawa H., Ugawa Y., Genba K., Shimpo T., Mannen T. (1988). Percutaneous electrical stimulation (PES) and SEP in peripheral neuropathies. Clin. Neurol..

[61] Xu X.Y., Yee W.C., Hwang P.Y.K., Yu H., Wan A.C.A., Gao S., Boon K.-L., Mao H.-Q., Leong K.W., Wang S. (2003). Peripheral nerve regeneration with sustained release of poly(phosphoester) microencapsulated nerve growth factor within nerve guide conduits. Biomaterials.

[62] Heine J., Schmiedl A., Cebotari S., Mertsching H., Karck M., Haverich A., Kallenbach K. (2011). Preclinical assessment of a tissue-engineered vasomotive human small-calibered vessel based on a decellularized xenogenic matrix: Histological and functional characterization. Tissue Eng. A..

[63] McCallister W.V., Tang P., Smith J., Trumble T.E. (2001). Axonal regeneration stimulated by the combination of nerve growth factor and ciliary neurotrophic factor in an end-to-side model. J. Hand Surg..

[64] Brown M.C., Perry V.H., Lunn E.R., Gordon S., Heumann R. (1991). Macrophage dependence of peripheral sensory nerve regeneration—Possible involvement of nerve growth-factor. Neuron.

[65] Lee J.Y., Bashur C.A., Goldstein A.S., Schmidt C.E. (2009). Polypyrrole-coated electrospun PLGA nanofibers for neural tissue applications. Biomaterials.

[66] Jiang X.P., Tessier D., Dao L.H., Zhang Z. (2002). Biostability of electrically conductive polyester fabrics: An *in vitro* study. J. Biomed. Mater. Res..

[67] Zawko S.A., Schmidt C.E. (2010). Simple benchtop patterning of hydrogel grids for living cell microarrays. Lab Chip.

[68] Fonner J.M., Forciniti L., Nguyen H., Byrne J.D., Kou Y.-F., Syeda-Nawaz J., Schmidt C.E. (2008). Biocompatibility implications of polypyrrole synthesis techniques. Biomed. Mater..

[69] Gordon T., Udina E., Verge V.M.K., de Chaves E.I.P. (2009). Brief electrical stimulation accelerates axon regeneration in the peripheral nervous system and promotes sensory axon regeneration in the central nervous system. Motor Contr..

[70] Gordon T., Udina E., Verge V.M.K., de Chaves E.I.P. (2010). (Correction) Brief electrical stimulation accelerates axon regeneration in the peripheral nervous system and promotes sensory axon regeneration in the central nervous system. Motor Contr..

[71] Al-Majed A.A., Neumann C.M., Brushart T.M., Gordon T. (2000). Brief electrical stimulation promotes the speed and accuracy of motor axonal regeneration. J. Neurosci..

[72] Brooks D.N., Weber R.V., Chao J.D., Rinker B.D., Zoldos J., Robichaux M.R., Ruggeri S.B., Anderson K.A., Bonatz E.E., Wisotsky S.M., Cho M.S., Wilson C., Cooper E.O., Ingari J.V., Safa B., Parrett B.M., Buncke G.M. (2012). Processed nerve allografts for peripheral nerve reconstruction: A multicenter study of utilization and outcomes in sensory, mixed, and motor nerve reconstructions. Microsurgery.

[73] Bechara S., Wadman L., Popat K.C. (2011). Electroconductive polymeric nanowire templates facilitates *in vitro* C17.2 neural stem cell line adhesion, proliferation and differentiation. Acta Biomater..

[74] Moroder P., Runge M.B., Wang H., Ruesink T., Lu L., Spinner R.J., Windebank A.J., Yaszemski M.J. (2011). Material properties and electrical stimulation regimens of polycaprolactone fumarate-polypyrrole scaffolds as potential conductive nerve conduits. Acta Biomater..

[75] Runge M.B., Dadsetan M., Baltrusaitis J., Knight A.M., Ruesink T., Lazcano E.A., Lu L., Windebank A.J., Yaszemski M.J. (2010). The development of electrically conductive polycaprolactone fumarate-polypyrrole composite materials for nerve regeneration. Biomaterials.

[76] Runge M.B., Dadsetan M., Baltrusaitis J., Ruesink T., Lu L., Windebank A.J., Yaszemski M.J. (2010). Development of electrically conductive oligo(polyethylene glycol) fumarate-polypyrrole hydrogels for nerve regeneration. Biomacromolecules.

[77] Runge M.B., Wang H., Spinner R.J., Windebank A.J., Yaszemski M.J. (2012). Reformulating polycaprolactone fumarate to eliminate toxic diethylene glycol: Effects of polymeric branching and autoclave sterilization on material properties. Acta Biomater..

[78] Eisazadeh H. (2007). Studying the characteristics of polypyrrole and its composites. World J. Chem..

[79] Bruno F.F., Fossey S.A., Nagarajan S., Nagarajan R., Kumar J., Samuelson L.A. (2006). Biomimetic synthesis of water-soluble conducting copolymers/homopolymers of pyrrole and 3,4-ethylenedioxythiophene. Biomacromolecules.

[80] Hardy J.G., Mouser D.J., Arroyo-Currás N., Geissler S., Chow J.K., Nguy L., Kim J.M., Schmidt C.E. (2014). Biodegradable electroactive polymers for electrochemically-triggered drug delivery. J. Mater. Chem. B.

[81] Bilston L.E. (2011). Neural Tissue Biomechanics.

[82] Guimard N.K.E., Sessler J.L., Schmidt C.E. (2009). Toward a biocompatible and biodegradable copolymer incorporating electroactive oligothiophene units. Macromolecules.

[83] Da Silva M.A., Crawford A., Mundy J., Martins A., Araújo J.V., Hatton P.V., Reis R.L., Neves N.M. (2009). Evaluation of extracellular matrix formation in polycaprolactone and starch-compounded polycaprolactone nanofiber meshes when seeded with bovine articular chondrocytes. Tissue Eng. A.

[84] Jukola H., Nikkola L., Gomes M.E., Chiellini F., Tukiainen M., Kellomäki M., Chiellini E., Reis R.L., Ashammakhi N. (2008). Development of a bioactive glass fiber reinforced starch-polycaprolactone composite. J. Biomed. Mater. Res. B.

[85] Woodruff M.A., Hutmacher D.W. (2010). The return of a forgotten polymer-polycaprolactone in the 21st century. Prog. Polym. Sci..

[86] Schmidt C.E., Shastri V.R., Vacanti J.P., Langer R. (1997). Stimulation of neurite outgrowth using an electrically conducting polymer. Proc. Nat. Acad. Sci. USA.

[87] Wang Z.X., Roberge C., Dao L.H., Wan Y., Shi G., Rouabhia M., Guidoin R., Zhang Z. (2004). *In vivo* evaluation of a novel electrically conductive polypyrrole/poly(D,L-lactide) composite and polypyrrole-coated-poly(D,L-lactide-co-glycolide) membranes. J. Biomed. Mater. Res. A.

[88] Mihardja S.S., Sievers R.E., Lee R.J. (2008). The effect of polypyrrole on arteriogenesis in an acute rat infarct model. Biomaterials.

[89] Durgam H., Sapp S., Deister C., Khaing Z., Chang E., Luebben S., Schmidt C.E. (2010). Novel degradable co-polymers of polypyrrole support cell proliferation and enhance neurite out-growth with electrical stimulation. J. Biomater. Sci. Polym. Ed..

[90] George P.M., Lyckman A.W., LaVan D.A., Hegde A., Leung Y., Avasare R., Testa C., Alexander P.M., Langer R., Sur M. (2005). Fabrication and biocompatibility of polypyrrole implants suitable for neural prosthetics. Biomaterials.

[91] Huang J., Ye Z., Hu X., Lu L., Luo Z. (2010). Electrical stimulation induces calcium-dependent release of NGF from cultured Schwann cells. Glia.

[92] Huang J., Hu X., Lu L., Ye Z., Zhang Q., Luo Z. (2010). Electrical regulation of Schwann cells using conductive polypyrrole/chitosan polymers. J. Biomed. Mater. Res. A.

[93] Zhu B., Luo S.C., Zhao H., Lin H.A., Sekine J., Nakao A., Chen C., Yamashita Y., Yu H. (2014). Large enhancement in neurite outgrowth on a cell membrane-mimicking conducting polymer. Nat. Commun..

[94] Koppes A.N., Nordberg A.L., Paolillo G.M., Goodsell N.M., Darwish H.A., Zhang L., Thompson D.M. (2014). Electrical stimulation of Schwann cells promotes sustained increases in neurite outgrowth. Tissue Eng. A.

[95] George P.M., LaVan D.A., Burdick J.A., Chen C.Y., Liang E., Langer R. (2006). Electrically controlled drug delivery from biotin-doped conductive polypyrrole. Adv. Mater..

[96] Cho Y., Shi R., Ivanisevic A., Ben Borgens R. (2009). A mesoporous silica nanosphere-based drug delivery system using an electrically conducting polymer. Nanotechnology.

[97] Liu J., Lamb D., Chou M.M., Liu Y., Li G. (2007). Nerve growth factor-mediated neurite outgrowth via regulation of Rab5. Mol Biol Cell..

[98] Hu Z.Q., Ulfendahl M., Olivius N.P. (2005). NGF stimulates extensive neurite outgrowth from implanted dorsal root ganglion neurons following transplantation into the adult rat inner ear. Neurobiol. Dis..

